# Supplementary material for: Improved lipidomic profile mediates the effects of adherence to healthy lifestyles on coronary heart disease
Source: eLife. 2021 Feb 9;10:e60999. doi: 10.7554/eLife.60999 (PMC7872516; doi:10.7554/eLife.60999)
Supplement: Figure 1—source data 1. [file elife-60999-fig1-data1.docx]

**Figure 1–Source Data 1. Associations of size-specific lipoprotein particle concentrations, mean lipoprotein particle diameter, and apolipoprotein concentrations with combined healthy lifestyle and risk of coronary heart disease.**

|  | **a) Adherence to combined healthy lifestyle vs metabolomics**  **(n=4,681)** | | | |  | **b) Metabolomics vs CHD**  **(n=2,440)** | |  | **Mediation effect of metabolomics** | |
| --- | --- | --- | --- | --- | --- | --- | --- | --- | --- | --- |
|  | **Adherence to 2-3 combined healthy lifestyles** | | **Adherence to 4-5 combined healthy lifestyles** | |  |  |  |  | **Proportion mediated, %** | **FDR** |
|  | **Beta (95% CI)** | **FDR** | **Beta (95% CI)** | **FDR** |  | **OR (95% CI)** | **FDR** |  |  |  |
| **Lipoprotein Particle Concentration** |  |  |  |  |  |  |  |  |  |  |
| **VLDL** |  |  |  |  |  |  |  |  |  | |
| Extremely large | -0.29 (-0.37, -0.22) | 1.80E-12 | -0.50 (-0.62, -0.39) | 3.83E-17 |  | 1.19 (1.08, 1.30) | 9.12E-04 |  | 6.08 | 0.030 |
| Very large | -0.30 (-0.38, -0.23) | 6.76E-13 | -0.53 (-0.64, -0.42) | 1.00E-18 |  | 1.20 (1.09, 1.32) | 5.08E-04 |  | 6.52 | 0.022 |
| Large | -0.30 (-0.38, -0.22) | 8.87E-13 | -0.54 (-0.66, -0.43) | 7.95E-19 |  | 1.22 (1.12, 1.35) | 8.44E-05 |  | 7.97 | 0.010 |
| Medium | -0.31 (-0.39, -0.23) | 7.92E-13 | -0.54 (-0.65, -0.42) | 1.00E-18 |  | 1.25 (1.14, 1.38) | 1.33E-05 |  | 9.07 | 0.006 |
| Small | -0.30 (-0.38, -0.22) | 1.22E-12 | -0.51 (-0.62, -0.39) | 2.24E-17 |  | 1.30 (1.18, 1.43) | 9.41E-07 |  | 10.15 | 0.005 |
| Very small | -0.20 (-0.28, -0.12) | 9.15E-07 | -0.27 (-0.38, -0.15) | 5.74E-06 |  | 1.27 (1.15, 1.40) | 6.84E-06 |  | 4.77 | 0.042 |
| **IDL** | -0.09 (-0.17, -0.02) | 2.30E-02 | -0.11 (-0.22, 0.00) | 7.33E-02 |  | 1.19 (1.08, 1.31) | 8.05E-04 |  | 1.17 | 0.502 |
| **LDL** |  |  |  |  |  |  |  |  |  | |
| Large | -0.08 (-0.16, 0.00) | 4.79E-02 | -0.11 (-0.22, 0.00) | 7.01E-02 |  | 1.20 (1.09, 1.32) | 5.78E-04 |  | 1.55 | 0.388 |
| Medium | -0.08 (-0.16, 0.00) | 5.06E-02 | -0.11 (-0.22, 0.00) | 5.47E-02 |  | 1.21 (1.01, 1.33) | 3.85E-04 |  | 1.88 | 0.301 |
| Small | -0.09 (-0.16, -0.01) | 3.33E-02 | -0.13 (-0.24, -0.02) | 2.34E-02 |  | 1.20 (1.09, 1.32) | 5.62E-04 |  | 2.16 | 0.223 |
| **HDL** |  |  |  |  |  |  |  |  |  | |
| Very large | 0.11 (0.03, 0.19) | 8.22E-03 | 0.20 (0.09, 0.31) | 7.64E-04 |  | 0.88 (0.80, 0.97) | 1.13E-02 |  | 2.67 | 0.130 |
| Large | 0.25 (0.17, 0.32) | 1.63E-09 | 0.39 (0.28, 0.50) | 2.77E-11 |  | 0.79 (0.72, 0.87) | 1.34E-05 |  | 8.02 | 0.008 |
| Medium | 0.12 (0.04, 0.20) | 6.19E-03 | 0.11 (-0.01, 0.22) | 8.48E-02 |  | 0.84 (0.77, 0.92) | 7.06E-04 |  | 1.22 | 0.439 |
| Small | -0.01 (-0.08, 0.07) | 9.07E-01 | -0.15 (-0.26, -0.04) | 1.44E-02 |  | 1.00 (0.91, 1.10) | 9.54E-01 |  | 0.10 | 0.909 |
| **Mean particle diameter** |  |  |  |  |  |  |  |  |  |  |
| VLDL | -0.25 (-0.33, -0.17) | 4.93E-09 | -0.47 (-0.59, -0.36) | 4.92E-15 |  | 1.14 (1.04, 1.25) | 8.87E-03 |  | 4.15 | 0.098 |
| LDL | 0.03 (-0.04, 0.10) | 4.64E-01 | 0.11 (0.01, 0.21) | 4.46E-02 |  | 0.97 (0.87, 1.08) | 6.25E-01 |  | 0.41 | 0.665 |
| HDL | 0.18 (0.11, 0.26) | 5.20E-06 | 0.32 (0.21, 0.43) | 2.07E-08 |  | 0.80 (0.73, 0.88) | 4.10E-05 |  | 6.84 | 0.011 |
| **Apolipoproteins** |  |  |  |  |  |  |  |  |  |  |
| Apolipoproteins A1 | 0.10 (0.02, 0.18) | 1.53E-02 | 0.11 (0.00, 0.23) | 7.25E-02 |  | 0.89 (0.81, 0.98) | 2.63E-02 |  | 1.27 | 0.295 |
| Apolipoproteins B | -0.25 (-0.33, -0.18) | 5.49E-10 | -0.40 (-0.51, -0.29) | 5.20E-12 |  | 1.30 (1.18, 1.43) | 1.78E-06 |  | 8.36 | 0.006 |
| Ratio: ApoB to ApoA1 | -0.30 (-0.38, -0.22) | 6.76E-13 | -0.45 (-0.56, -0.34) | 1.32E-14 |  | 1.35 (1.23, 1.49) | 3.43E-08 |  | 11.32 | <0.001 |

CHD = coronary heart disease; OR = odds ratio; CI = confidence interval; FDR = false discovery rate; VLDL = very low-density lipoprotein; IDL = intermediate-density lipoprotein; LDL = low-density lipoprotein; HDL = high-density lipoprotein. a) Beta and 95% CI are for comparison of participants who adopted 2-3 or 4-5 healthy lifestyles with participants who adopted 0-1. Multivariable model was adjusted for: age, sex, fasting time, study sites, education level, and case/control status. b) Odds ratio and 95% CI are for the associations of 1-SD metabolic markers increasing with CHD risk. Multivariable model was adjusted for: age, sex, fasting time, study sites, education level, and smoking status.
