# Supplementary material for: Improved lipidomic profile mediates the effects of adherence to healthy lifestyles on coronary heart disease
Source: eLife. 2021 Feb 9;10:e60999. doi: 10.7554/eLife.60999 (PMC7872516; doi:10.7554/eLife.60999)
Supplement: Figure 2—source data 1. [file elife-60999-fig2-data1.docx]

**Figure 2–Source Data 1. Associations of** **cholesterol concentrations in lipoprotein subfractions with combined healthy lifestyle and risk of coronary heart disease.**

|  | **a) Adherence to combined healthy lifestyle vs metabolomics**  **(n=4,681)** | | | | |  | **b) Metabolomics vs CHD**  **(n=2,440)** | |  | **Mediation effect of metabolomics** | |
| --- | --- | --- | --- | --- | --- | --- | --- | --- | --- | --- | --- |
|  | **Adherence to 2-3 combined healthy lifestyles** | | | **Adherence to 4-5 combined healthy lifestyles** | |  |  |  |  | **Proportion mediated, %** | **FDR** |
|  | **Beta (95% CI)** | | **FDR** | **Beta (95% CI)** | **FDR** |  | **OR (95% CI)** | **FDR** |  |  |  |
| **Total** | | -0.05 (-0.13, 0.02) | 2.09E-01 | -0.08 (-0.19, 0.03) | 1.77E-01 |  | 1.13 (1.03, 1.25) | 1.64E-02 |  | 0.63 | 0.591 |
| **VLDL** | | -0.29 (-0.37, -0.21) | 2.05E-12 | -0.47 (-0.58, -0.36) | 2.00E-15 |  | 1.28 (1.16, 1.41) | 3.52E-06 |  | 8.73 | 0.006 |
| **Remnant** | | -0.23 (-0.31, -0.15) | 1.77E-08 | -0.35 (-0.47, -0.24) | 1.18E-09 |  | 1.27 (1.15, 1.40) | 7.67E-06 |  | 6.37 | 0.013 |
| **LDL** | | -0.05 (-0.12, 0.03) | 2.87E-01 | -0.06 (-0.17, 0.05) | 3.46E-01 |  | 1.16 (1.06, 1.28) | 3.30E-03 |  | 0.95 | 0.521 |
| **HDL** | | 0.24 (0.16, 0.32) | 8.30E-09 | 0.35 (0.23, 0.46) | 6.58E-09 |  | 0.80 (0.73, 0.88) | 1.08E-05 |  | 6.51 | 0.011 |
| HDL2 | | 0.25 (0.17, 0.32) | 4.65E-09 | 0.36 (0.24, 0.47) | 2.74E-09 |  | 0.79 (0.72, 0.87) | 4.42E-06 |  | 6.92 | 0.010 |
| HDL3 | | 0.13 (0.06, 0.21) | 1.40E-03 | 0.17 (0.05, 0.28) | 5.50E-03 |  | 0.96 (0.88, 1.06) | 5.03E-01 |  | 0.18 | 0.907 |
| **Esterified** | | -0.04 (-0.11, 0.04) | 4.05E-01 | -0.06 (-0.17, 0.05) | 3.37E-01 |  | 1.12 (1.02, 1.23) | 3.08E-02 |  | 0.53 | 0.625 |
| **Free** | | -0.10 (-0.18, -0.02) | 1.80E-02 | -0.13 (-0.24, -0.02) | 3.20E-02 |  | 1.15 (1.04, 1.26) | 7.01E-03 |  | 0.96 | 0.487 |
| **VLDL** | |  |  |  |  |  |  |  |  |  | |
| Extremely large | | -0.30 (-0.38, -0.22) | 7.07E-13 | -0.51 (-0.62, -0.40) | 1.19E-17 |  | 1.23 (1.12, 1.35) | 9.35E-05 |  | 7.25 | 0.013 |
| Very large | | -0.30 (-0.38, -0.22) | 6.76E-13 | -0.52 (-0.63, -0.41) | 1.97E-18 |  | 1.21 (1.10, 1.33) | 2.29E-04 |  | 6.76 | 0.017 |
| Large | | -0.30 (-0.38, -0.22) | 8.51E-13 | -0.53 (-0.65, -0.42) | 1.05E-18 |  | 1.23 (1.12, 1.35) | 5.59E-05 |  | 7.90 | 0.010 |
| Medium | | -0.30 (-0.38, -0.22) | 1.22E-12 | -0.52 (-0.63, -0.40) | 1.05E-17 |  | 1.27 (1.15, 1.39) | 5.94E-06 |  | 8.91 | 0.006 |
| Small | | -0.20 (-0.28, -0.13) | 7.32E-07 | -0.32 (-0.43, -0.21) | 4.92E-08 |  | 1.27 (1.16, 1.40) | 5.72E-06 |  | 5.85 | 0.018 |
| Very small | | -0.10 (-0.18, -0.02) | 1.51E-02 | -0.09 (-0.20, 0.02) | 1.21E-01 |  | 1.16 (1.05, 1.28) | 4.42E-03 |  | 0.86 | 0.523 |
| **IDL** | | -0.05 (-0.13, 0.03) | 2.55E-01 | -0.05 (-0.16, 0.06) | 4.13E-01 |  | 1.15 (1.04, 1.26) | 8.63E-03 |  | 0.54 | 0.657 |
| **LDL** | |  |  |  |  |  |  |  |  |  | |
| Large | | -0.05 (-0.13, 0.03) | 2.52E-01 | -0.06 (-0.17, 0.05) | 3.18E-01 |  | 1.17 (1.06, 1.29) | 2.88E-03 |  | 0.95 | 0.521 |
| Medium | | -0.04 (-0.11, 0.04) | 3.79E-01 | -0.05 (-0.16, 0.06) | 4.23E-01 |  | 1.17 (1.06, 1.28) | 2.88E-03 |  | 1.04 | 0.499 |
| Small | | -0.03 (-0.10, 0.05) | 5.16E-01 | -0.03 (-0.14, 0.08) | 5.83E-01 |  | 1.15 (1.05, 1.27) | 6.25E-03 |  | 0.84 | 0.523 |
| **HDL** | |  |  |  |  |  |  |  |  |  | |
| Very large | | 0.04 (-0.04, 0.12) | 3.08E-01 | 0.08 (-0.04, 0.19) | 2.10E-01 |  | 0.91 (0.82, 1.00) | 5.99E-02 |  | 1.04 | 0.354 |
| Large | | 0.25 (0.17, 0.33) | 1.02E-09 | 0.40 (0.29, 0.51) | 9.71E-12 |  | 0.78 (0.71, 0.86) | 5.25E-06 |  | 8.69 | 0.006 |
| Medium | | 0.18 (0.10, 0.26) | 2.73E-05 | 0.21 (0.10, 0.33) | 4.27E-04 |  | 0.80 (0.73, 0.88) | 9.62E-06 |  | 3.18 | 0.124 |
| Small | | 0.16 (0.08, 0.23) | 8.35E-05 | 0.17 (0.06, 0.28) | 2.98E-03 |  | 1.00 (0.91, 1.11) | 9.64E-01 |  | -0.17 | 0.742 |

CHD = coronary heart disease; OR = odds ratio; CI = confidence interval; FDR = false discovery rate; VLDL = very low-density lipoprotein; IDL = intermediate-density lipoprotein; LDL = low-density lipoprotein; HDL = high-density lipoprotein. a) Beta and 95% CI are for comparison of participants who adopted 2-3 or 4-5 combined healthy lifestyles with participants who adopted 0-1. Multivariable model was adjusted for: age, sex, fasting time, study areas, education level, and case/control status. b) Odds ratio and 95% CI are for the associations of 1-SD metabolic markers increasing with CHD risk. Multivariable model was adjusted for: age, sex, fasting time, study areas, education level, and smoking status.
