# Supplementary material for: Improved lipidomic profile mediates the effects of adherence to healthy lifestyles on coronary heart disease
Source: eLife. 2021 Feb 9;10:e60999. doi: 10.7554/eLife.60999 (PMC7872516; doi:10.7554/eLife.60999)
Supplement: Figure 3—source data 1. [file elife-60999-fig3-data1.docx]

**Figure 3–Source Data 1. Associations of triglyceride concentrations in lipoprotein subfractions with combined healthy lifestyle and risk of coronary heart disease.**

| **Triglycerides Concentration** | **a) Adherence to combined healthy lifestyle to metabolomics**  **(n=4,681)** | | | |  | **b) Metabolomics vs CHD**  **(n=2,440)** | |  | **Mediation effect of metabolomics** | |
| --- | --- | --- | --- | --- | --- | --- | --- | --- | --- | --- |
|  | **Adherence to 2-3 combined healthy lifestyles** | | **Adherence to 4-5 combined healthy lifestyles** | |  |  |  |  | **Proportion mediated, %** | **FDR** |
|  | **Beta (95% CI)** | **FDR** | **Beta (95% CI)** | **FDR** |  | **OR (95% CI)** | **FDR** |  |  |  |
| **Total** | -0.32 (-0.40, -0.24) | 4.58E-13 | -0.56 (-0.67, -0.44) | 7.95E-19 |  | 1.27 (1.15, 1.39) | 5.72E-06 |  | 9.86 | 0.006 |
| **VLDL** | -0.31 (-0.39, -0.23) | 5.47E-13 | -0.55 (-0.66, -0.43) | 7.95E-19 |  | 1.24 (1.13, 1.37) | 2.28E-05 |  | 8.90 | 0.008 |
| **LDL** | -0.23 (-0.31, -0.15) | 1.71E-08 | -0.35 (-0.46, -0.24) | 2.89E-09 |  | 1.34 (1.21, 1.48) | 2.01E-07 |  | 7.11 | 0.018 |
| **HDL** | -0.23 (-0.31, -0.16) | 1.47E-08 | -0.39 (-0.50, -0.28) | 3.69E-11 |  | 1.22 (1.11, 1.34) | 1.65E-04 |  | 4.37 | 0.041 |
| **VLDL** |  |  |  |  |  |  |  |  |  | |
| Extremely large | -0.29 (-0.37, -0.21) | 3.46E-12 | -0.50 (-0.61, -0.39) | 4.58E-17 |  | 1.18 (1.08, 1.30) | 9.76E-04 |  | 6.11 | 0.030 |
| Very large | -0.30 (-0.38, -0.23) | 6.76E-13 | -0.53 (-0.64, -0.42) | 1.00E-18 |  | 1.19 (1.08, 1.31) | 6.62E-04 |  | 6.38 | 0.023 |
| Large | -0.30 (-0.38, -0.22) | 1.22E-12 | -0.54 (-0.66, -0.43) | 7.95E-19 |  | 1.22 (1.11, 1.34) | 1.13E-04 |  | 7.86 | 0.011 |
| Medium | -0.30 (-0.38, -0.22) | 8.51E-13 | -0.54 (-0.65, -0.42) | 1.00E-18 |  | 1.24 (1.13, 1.37) | 2.48E-05 |  | 8.82 | 0.008 |
| Small | -0.31 (-0.39, -0.23) | 5.26E-13 | -0.55 (-0.66, -0.43) | 7.95E-19 |  | 1.29 (1.17, 1.42) | 1.80E-06 |  | 10.47 | 0.006 |
| Very small | -0.30 (-0.38, -0.22) | 1.25E-12 | -0.49 (-0.61, -0.38) | 2.17E-16 |  | 1.32 (1.21, 1.46) | 2.01E-07 |  | 10.29 | 0.006 |
| **IDL** | -0.24 (-0.32, -0.16) | 4.09E-09 | -0.37 (-0.49, -0.26) | 1.96E-10 |  | 1.37 (1.24, 1.52) | 2.83E-08 |  | 8.80 | 0.010 |
| **LDL** |  |  |  |  |  |  |  |  |  | |
| Large | -0.21 (-0.28, -0.13) | 4.37E-07 | -0.32 (-0.43, -0.21) | 5.64E-08 |  | 1.35 (1.22, 1.49) | 1.74E-07 |  | 6.17 | 0.039 |
| Medium | -0.20 (-0.28, -0.12) | 1.03E-06 | -0.29 (-0.41, -0.18) | 6.53E-07 |  | 1.33 (1.21, 1.47) | 2.03E-07 |  | 4.65 | 0.102 |
| Small | -0.28 (-0.36, -0.20) | 1.07E-11 | -0.46 (-0.57, -0.34) | 2.03E-14 |  | 1.33 (1.21, 1.46) | 2.01E-07 |  | 8.58 | 0.008 |
| **HDL** |  |  |  |  |  |  |  |  |  | |
| Very large | -0.21 (-0.28, -0.13) | 3.97E-07 | -0.32 (-0.43, -0.21) | 3.76E-08 |  | 1.17 (1.06, 1.29) | 4.14E-03 |  | 2.31 | 0.148 |
| Large | 0.04 (-0.03, 0.11) | 3.05E-01 | 0.08 (-0.03, 0.19) | 1.57E-01 |  | 1.03 (0.93, 1.14) | 6.22E-01 |  | -0.85 | 0.531 |
| Medium | -0.23 (-0.31, -0.15) | 5.56E-08 | -0.41 (-0.52, -0.29) | 1.75E-11 |  | 1.19 (1.09, 1.31) | 5.08E-04 |  | 4.79 | 0.031 |
| Small | -0.30 (-0.38, -0.22) | 2.26E-12 | -0.53 (-0.65, -0.42) | 1.37E-18 |  | 1.28 (1.16, 1.40) | 2.99E-06 |  | 9.02 | 0.006 |

CHD = coronary heart disease; OR = odds ratio; CI = confidence interval; FDR = false discovery rate; VLDL = very low-density lipoprotein; IDL = intermediate-density lipoprotein; LDL = low-density lipoprotein; HDL = high-density lipoprotein. a) Beta and 95% CI are for comparison of participants who adopted 2-3 or 4-5 combined healthy lifestyles with participants who adopted 0-1. Multivariable model was adjusted for: age, sex, fasting time, study areas, education level, and case/control status. b) Odds ratio and 95% CI are for the associations of 1-SD metabolic markers increasing with CHD risk. Multivariable model was adjusted for: age, sex, fasting time, study areas, education level, and smoking status.
