# Supplementary material for: Improved lipidomic profile mediates the effects of adherence to healthy lifestyles on coronary heart disease
Source: eLife. 2021 Feb 9;10:e60999. doi: 10.7554/eLife.60999 (PMC7872516; doi:10.7554/eLife.60999)
Supplement: Supplementary file 2. — (A) Age-, sex-, and study area-adjusted baseline characteristics of 4681 participants according to case or control status. (B) Sensitivity analysis of association between combined healthy lifestyle and lipid metabolites. (C) Associations of the HMGCR score with changes in the lipid metabolites, and subgroup analysis of the association between healthy lifestyle and lipid metabolites according to HMGCR score. (D) Associations of the ACLY score with changes in the lipid metabolites, and subgroup analysis of the association between healthy lifestyle and lipid metabolites according to ACLY score. (E) Associations of the sum of HMGCR and ACLY score with changes in the lipid metabolites, and subgroup analysis of the association between healthy lifestyle and lipid metabolites according to the sum score. (F) Associations of the genetic scores* with changes in the lipid metabolites. (G) Food frequency questionnaire used in the CKB study at baseline. (H) Variants included in the genetic scores and their associations with plasma low-density lipoprotein cholesterol. [file elife-60999-supp2.docx]

**Supplementary file 2A. Age-, sex-, and study area-adjusted baseline characteristics of 4,681 participants according to case or control status**

| **Baseline characteristics** | **Coronary heart**  **disease**  **cases** | **Ischemic Stroke**  **cases** | **Intracerebral**  **Hemorrhage**  **cases** | **Controls** |
| --- | --- | --- | --- | --- |
| No. of participants, n (%) | 927  (19.8) | 1,114  (23.8) | 1,127  (24.1) | 1,513  (32.3) |
| Age, year | 52.5 | 43.0 | 47.4 | 45.4 |
| Female, % | 39.0 | 55.7 | 51.3 | 52.3 |
| Urban area, % | 29.0 | 40.0 | 23.7 | 25.0 |
| Middle school and above, % | 54.6 | 55.4 | 57.1 | 55.5 |
| Married, % | 93.5 | 94.0 | 94.5 | 95.6 |
| Prevalent hypertension, % | 46.7 | 47.5 | 66.4 | 27.3 |
| Prevalent diabetes, % | 9.1 | 8.1 | 6.6 | 3.2 |
| Family history of heart attack, % | 5.9 | 4.2 | 3.9 | 4.3 |
| Having healthy lifestyle factors*, % |  |  |  |  |
| Never smoking | 57.4 | 59.1 | 61.3 | 62.5 |
| Moderate alcohol consumption | 8.5 | 11.4 | 11.9 | 12.3 |
| Physical activity | 47.2 | 50.2 | 50.6 | 51.1 |
| Healthy dietary pattern | 48.3 | 50.9 | 46.9 | 50.3 |
| Vegetables 7 days/week | 93.8 | 92.7 | 92.9 | 93.0 |
| Fruit 7 days/week | 12.5 | 13.2 | 11.2 | 15.3 |
| Read meat < 7 days/week | 74.2 | 72.6 | 74.2 | 75.6 |
| Soybean product ≥ 4 days/week | 7.5 | 9.9 | 7.9 | 9.7 |
| Fish ≥ 1 days/week | 32.3 | 36.7 | 31.7 | 35.3 |
| Coarse grains ≥ 4 days/week | 22.3 | 22.7 | 22.8 | 24.1 |
| Healthy adiposity level | 65.6 | 64.5 | 66.0 | 74.8 |
| Fasting time, h | 4.2 | 4.5 | 4.2 | 4.1 |

The results are presented as adjusted means or percentages, with adjustment for age, sex, and study area, as appropriate.

*Healthy lifestyle factors were defined as: never smoking; weekly but not daily drinking or daily drinking less than 30 g of pure alcohol per day; engaging in a sex-specific median or higher level of physical activity; engaging in more than or equal to 4 of total 6 healthy diet components; having a body mass index between 18.5 and 27.9 kg/m2 and having a waist circumference < 90 in men and < 85 centimeter in women.

# Supplementary file 2B. Sensitivity analysis of association between combined healthy lifestyle and lipid metabolites

| **Metabolomics** | **Basic model* + adjusted for prevalent diabetes**  **(n=4,681)** | | | |  | **Basic model* in the control participants**  **(n=1,513)** | | | | | | | | |
| --- | --- | --- | --- | --- | --- | --- | --- | --- | --- | --- | --- | --- | --- | --- |
|  | **Adherence to 2-3 healthy lifestyles** | | **Adherence to 4-5 healthy lifestyles** | |  | **Adherence to 2-3 healthy lifestyles** | | **Adherence to 4-5**  **healthy lifestyles** | | | | | | |
|  | **Beta**  **(95% CI)** | **FDR** | **Beta**  **(95% CI)** | **FDR** |  | **Beta**  **(95% CI)** | **FDR** | | **Beta**  **(95% CI)** | **FDR** | | | | |
| **Lipoprotein Particle Concentration** |  |  |  |  |  |  |  | |  |  | | | | |
| **VLDL** |  |  |  |  |  |  |  | |  |  | | | | |
| Extremely large | -0.26  (-0.34, -0.18) | 7.11E-10 | -0.46  (-0.57, -0.35) | 1.31E-14 |  | -0.35  (-0.51, -0.20) | 5.80E-05 | | -0.42  (-0.63, -0.22) | | | 3.36E-04 | | |
| Very large | -0.27  (-0.35, -0.19) | 2.93E-10 | -0.48  (-0.60, -0.37) | 5.98E-16 |  | -0.36  (-0.51, -0.20) | 5.80E-05 | | -0.43  (-0.63, -0.23) | | | 3.35E-04 | | |
| Large | -0.26  (-0.34, -0.18) | 5.11E-10 | -0.49  (-0.61, -0.38) | 5.60E-16 |  | -0.34  (-0.49, -0.19) | 9.03E-05 | | -0.45  (-0.65, -0.24) | | | 3.25E-04 | | |
| Medium | -0.27  (-0.35, -0.19) | 4.25E-10 | -0.49  (-0.60, -0.38) | 5.98E-16 |  | -0.35  (-0.50, -0.20) | 5.99E-05 | | -0.44  (-0.64, -0.24) | | | 3.25E-04 | | |
| Small | -0.26  (-0.34, -0.18) | 6.71E-10 | -0.46  (-0.57, -0.35) | 1.23E-14 |  | -0.34  (-0.49, -0.19) | 7.04E-05 | | -0.41  (-0.61, -0.21) | | | 3.71E-04 | | |
| Very small | -0.18  (-0.25, -0.10) | 1.83E-05 | -0.24  (-0.35, -0.13) | 6.03E-05 |  | -0.22  (-0.37, -0.07) | 1.13E-02 | | -0.19  (-0.39, 0.01) | | | 1.17E-01 | | |
| **IDL** | -0.09  (-0.16, -0.01) | 3.96E-02 | -0.10  (-0.21, 0.01) | 1.05E-01 |  | -0.10  (-0.25, 0.05) | 2.48E-01 | | -0.06  (-0.25, 0.14) | | | 6.44E-01 | | |
| **LDL** |  |  |  |  |  |  |  | |  | | |  | | |
| Large | -0.07  (-0.15, 0.00) | 7.71E-02 | -0.10  (-0.21, 0.01) | 9.94E-02 |  | -0.09  (-0.24, 0.05) | 3.00E-01 | | -0.07  (-0.26, 0.13) | | | 5.93E-01 | | |
| Medium | -0.07  (-0.15, 0.00) | 8.52E-02 | -0.10  (-0.21, 0.01) | 8.31E-02 |  | -0.08  (-0.23, 0.06) | 3.53E-01 | | -0.07  (-0.27, 0.12) | | | 5.62E-01 | | |
| Small | -0.08  (-0.15, 0.00) | 6.54E-02 | -0.12  (-0.23, -0.01) | 4.30E-02 |  | -0.09  (-0.23, 0.06) | 3.36E-01 | | -0.09  (-0.28, 0.10) | | | 4.72E-01 | | |
| **HDL** |  |  |  |  |  |  |  | |  | | |  | | |
| Very large | 0.11  (0.03, 0.18) | 1.35E-02 | 0.19  (0.08, 0.31) | 1.29E-03 |  | 0.13  (-0.03, 0.28) | 1.54E-01 | | 0.18  (-0.02, 0.38) | | | 1.41E-01 | | |
| Large | 0.23  (0.15, 0.31) | 2.31E-08 | 0.37  (0.26, 0.48) | 3.17E-10 |  | 0.30  (0.15, 0.44) | 3.22E-04 | | 0.36  (0.17, 0.55) | | | 1.21E-03 | | |
| Medium | 0.12  (0.04, 0.20) | 4.46E-03 | 0.11  (0.00, 0.23) | 7.01E-02 |  | 0.13  (-0.02, 0.28) | 1.38E-01 | | 0.08  (-0.12, 0.28) | | | 5.29E-01 | | |
| Small | 0.01  (-0.06, 0.09) | 7.46E-01 | -0.12  (-0.24, -0.01) | 4.30E-02 |  | 0.00  (-0.15, 0.15) | 9.93E-01 | | -0.13  (-0.33, 0.07) | | | 3.08E-01 | | |
| **Cholesterol Concentration** |  |  |  |  |  |  |  | |  | | |  | | |
| **VLDL** |  |  |  |  |  |  |  | |  | | |  | | |
| Extremely large | -0.27  (-0.34, -0.19) | 2.93E-10 | -0.47  (-0.58, -0.35) | 3.98E-15 |  | -0.36  (-0.52, -0.21) | 5.80E-05 | | -0.41  (-0.62, -0.21) | | | 3.64E-04 | | |
| Very large | -0.27  (-0.35, -0.19) | 2.93E-10 | -0.48  (-0.59, -0.37) | 7.81E-16 |  | -0.36  (-0.51, -0.21) | 5.80E-05 | | -0.42  (-0.62, -0.22) | | | 3.43E-04 | | |
| Large | -0.27  (-0.34, -0.19) | 4.25E-10 | -0.49  (-0.60, -0.38) | 5.98E-16 |  | -0.34  (-0.50, -0.19) | 7.96E-05 | | -0.43  (-0.64, -0.23) | | | 3.29E-04 | | |
| Medium | -0.26  (-0.34, -0.18) | 5.51E-10 | -0.47  (-0.59, -0.36) | 3.98E-15 |  | -0.35  (-0.51, -0.20) | 5.99E-05 | | -0.41  (-0.62, -0.21) | | | 3.71E-04 | | |
| Small | -0.18  (-0.25, -0.10) | 2.07E-05 | -0.29  (-0.40, -0.18) | 1.08E-06 |  | -0.22  (-0.37, -0.08) | 8.06E-03 | | -0.23  (-0.42, -0.03) | | | 4.93E-02 | | |
| Very small | -0.09  (-0.17, -0.01) | 2.82E-02 | -0.08  (-0.19, 0.03) | 1.71E-01 |  | -0.11  (-0.26, 0.04) | 2.16E-01 | | -0.04  (-0.24, 0.15) | | | 7.23E-01 | | |
| **IDL** | -0.05  (-0.12, 0.03) | 2.85E-01 | -0.05  (-0.16, 0.06) | 4.38E-01 |  | -0.07  (-0.21, 0.08) | 4.57E-01 | | -0.03  (-0.22, 0.17) | | | 8.20E-01 | | |
| **LDL** |  |  |  |  |  |  |  | |  | | |  | | |
| Large | -0.05  (-0.12, 0.03) | 2.91E-01 | -0.06  (-0.17, 0.05) | 3.52E-01 |  | -0.06  (-0.21, 0.09) | 4.98E-01 | | -0.04  (-0.23, 0.16) | | | 7.60E-01 | | |
| Medium | -0.03  (-0.11, 0.04) | 4.24E-01 | -0.04  (-0.16, 0.07) | 4.58E-01 |  | -0.04  (-0.19, 0.10) | 6.40E-01 | | -0.03  (-0.23, 0.16) | | | 7.92E-01 | | |
| Small | -0.03  (-0.10, 0.05) | 5.49E-01 | -0.03  (-0.14, 0.08) | 6.22E-01 |  | -0.03  (-0.18, 0.12) | 7.55E-01 | | -0.02  (-0.22, 0.17) | | | 8.33E-01 | | |
| **HDL** |  |  |  |  |  |  |  | |  | | |  | | |
| Very large | 0.04  (-0.04, 0.12) | 3.19E-01 | 0.08  (-0.04, 0.19) | 2.13E-01 |  | 0.03  (-0.13, 0.18) | 7.80E-01 | | 0.06  (-0.14, 0.26) | | | 6.56E-01 | | |
| Large | 0.23  (0.15, 0.31) | 1.95E-08 | 0.37  (0.26, 0.49) | 1.46E-10 |  | 0.29  (0.15, 0.44) | 3.46E-04 | | 0.36  (0.16, 0.55) | | | 1.28E-03 | | |
| Medium | 0.17  (0.09, 0.25) | 5.09E-05 | 0.21  (0.09, 0.32) | 6.22E-04 |  | 0.19  (0.04, 0.34) | 2.95E-02 | | 0.15  (-0.05, 0.35) | | | 2.14E-01 | | |
| Small | 0.15  (0.08, 0.23) | 1.86E-04 | 0.17  (0.06, 0.28) | 4.83E-03 |  | 0.20  (0.05, 0.34) | 1.86E-02 | | 0.15  (-0.05, 0.34) | | | 2.19E-01 | | |
| **Triglycerides Concentration** |  |  |  |  |  |  |  | |  | | |  | | |
| **VLDL** |  |  |  |  |  |  |  | |  | | |  | | |
| Extremely large | -0.25  (-0.33, -0.18) | 1.18E-09 | -0.46  (-0.57, -0.34) | 1.47E-14 |  | -0.35  (-0.50, -0.20) | 6.14E-05 | | -0.42  (-0.63, -0.22) | | | 3.36E-04 | | |
| Very large | -0.27  (-0.34, -0.19) | 3.03E-10 | -0.48  (-0.60, -0.37) | 5.98E-16 |  | -0.35  (-0.51, -0.20) | 5.80E-05 | | -0.43  (-0.63, -0.23) | | | 3.29E-04 | | |
| Large | -0.26  (-0.34, -0.18) | 6.63E-10 | -0.49  (-0.61, -0.38) | 5.60E-16 |  | -0.34  (-0.49, -0.18) | 1.05E-04 | | -0.45  (-0.65, -0.25) | | | 3.25E-04 | | |
| Medium | -0.26  (-0.34, -0.19) | 5.11E-10 | -0.49  (-0.60, -0.38) | 5.98E-16 |  | -0.34  (-0.50, -0.19) | 7.04E-05 | | -0.45  (-0.65, -0.24) | | | 3.25E-04 | | |
| Small | -0.27  (-0.35, -0.19) | 2.93E-10 | -0.50  (-0.61, -0.38) | 5.60E-16 |  | -0.35  (-0.51, -0.20) | 5.80E-05 | | -0.45  (-0.65, -0.25) | | | 3.25E-04 | | |
| Very small | -0.26  (-0.34, -0.18) | 7.11E-10 | -0.44  (-0.56, -0.33) | 1.05E-13 |  | -0.32  (-0.47, -0.16) | 2.58E-04 | | -0.37  (-0.57, -0.17) | | | 1.28E-03 | | |
| **IDL** | -0.21  (-0.29, -0.13) | 4.24E-07 | -0.33  (-0.45, -0.22) | 1.36E-08 |  | -0.26  (-0.41, -0.11) | 2.36E-03 | | -0.30  (-0.50, -0.10) | | | 1.01E-02 | | |
| **LDL** |  |  |  |  |  |  |  | |  | | |  | | |
| Large | -0.18  (-0.25, -0.10) | 1.83E-05 | -0.28  (-0.39, -0.17) | 1.66E-06 |  | -0.23  (-0.38, -0.08) | 8.05E-03 | | -0.24  (-0.44, -0.04) | | | 4.23E-02 | | |
| Medium | -0.17  (-0.25, -0.10) | 3.12E-05 | -0.26  (-0.37, -0.15) | 1.21E-05 |  | -0.17  (-0.33, -0.02) | 4.59E-02 | | -0.18  (-0.38, 0.03) | | | 1.45E-01 | | |
| Small | -0.25  (-0.32, -0.17) | 3.68E-09 | -0.41  (-0.52, -0.30) | 4.72E-12 |  | -0.26  (-0.41, -0.10) | 2.72E-03 | | -0.31  (-0.51, -0.10) | | | 8.49E-03 | | |
| **HDL** |  |  |  |  |  |  |  | |  | | |  | | |
| Very large | -0.18  (-0.26, -0.10) | 9.82E-06 | -0.29  (-0.40, -0.18) | 7.06E-07 |  | -0.28  (-0.42, -0.13) | 7.48E-04 | | -0.21  (-0.41, -0.02) | | | 6.21E-02 | | |
| Large | 0.04  (-0.03, 0.12) | 2.79E-01 | 0.08  (-0.02, 0.19) | 1.40E-01 |  | 0.06  (-0.08, 0.20) | 4.80E-01 | | 0.16  (-0.03, 0.34) | | | 1.58E-01 | | |
| Medium | -0.19  (-0.27, -0.12) | 4.59E-06 | -0.36  (-0.48, -0.25) | 1.62E-09 |  | -0.23  (-0.39, -0.08) | 8.06E-03 | | -0.30  (-0.50, -0.09) | | | 1.08E-02 | | |
| Small | -0.26  (-0.34, -0.18) | 1.17E-09 | -0.49  (-0.60, -0.37) | 7.81E-16 |  | -0.31  (-0.46, -0.15) | 4.16E-04 | | -0.41  (-0.62, -0.20) | | | 5.35E-04 | | |
| **Mean particle diameter** |  |  |  |  |  |  |  |  | |  | | |  |  |
| VLDL | -0.22  (-0.30, -0.14) | 3.79E-07 | -0.43  (-0.55, -0.32) | 6.06E-13 |  | -0.27  (-0.42, -0.11) | 2.32E-03 | | -0.39  (-0.6, -0.18) | | 1.02E-03 | | |  |
| LDL | 0.02  (-0.05, 0.09) | 6.77E-01 | 0.09  (-0.01, 0.19) | 8.58E-02 |  | 0.02  (-0.12, 0.16) | 8.45E-01 | | 0.11  (-0.07, 0.29) | | 3.42E-01 | | |  |
| HDL | 0.17  (0.09, 0.25) | 3.72E-05 | 0.31  (0.19, 0.42) | 1.49E-07 |  | 0.22  (0.07, 0.37) | 8.92E-03 | | 0.29  (0.09, 0.48) | | 1.08E-02 | | |  |
| **Cholesterol concentration** |  |  |  |  |  |  |  | |  | |  | | |  |
| Total | -0.04  (-0.12, 0.03) | 3.06E-01 | -0.07  (-0.18, 0.04) | 2.46E-01 |  | -0.06  (-0.21, 0.08) | 4.78E-01 | | -0.04  (-0.24, 0.15) | | 7.25E-01 | | |  |
| VLDL | -0.26  (-0.34, -0.18) | 7.11E-10 | -0.43  (-0.54, -0.32) | 4.55E-13 |  | -0.35  (-0.51, -0.20) | 5.80E-05 | | -0.37  (-0.57, -0.17) | | 1.12E-03 | | |  |
| Remnant | -0.20  (-0.28, -0.13) | 7.09E-07 | -0.32  (-0.43, -0.21) | 3.18E-08 |  | -0.29  (-0.43, -0.14) | 5.40E-04 | | -0.27  (-0.46, -0.07) | | 1.74E-02 | | |  |
| LDL | -0.04  (-0.12, 0.03) | 3.20E-01 | -0.05  (-0.16, 0.06) | 3.80E-01 |  | -0.05  (-0.20, 0.10) | 6.07E-01 | | -0.03  (-0.23, 0.16) | | 7.92E-01 | | |  |
| HDL | 0.23  (0.15, 0.31) | 7.14E-08 | 0.33  (0.22, 0.44) | 3.96E-08 |  | 0.29  (0.14, 0.44) | 6.23E-04 | | 0.30  (0.10, 0.50) | | 8.17E-03 | | |  |
| HDL2 | 0.23  (0.15, 0.31) | 4.75E-08 | 0.34  (0.22, 0.45) | 1.97E-08 |  | 0.29  (0.14, 0.44) | 5.54E-04 | | 0.30  (0.10, 0.50) | | 8.17E-03 | | |  |
| HDL3 | 0.13  (0.06, 0.21) | 1.45E-03 | 0.17  (0.05, 0.28) | 5.45E-03 |  | 0.17  (0.02, 0.32) | 4.37E-02 | | 0.23  (0.03, 0.43) | | 4.65E-02 | | |  |
| Esterified | -0.03  (-0.11, 0.05) | 5.04E-01 | -0.05  (-0.16, 0.06) | 4.17E-01 |  | -0.08  (-0.23, 0.07) | 3.69E-01 | | -0.05  (-0.24, 0.15) | | 7.18E-01 | | |  |
| Free | -0.08  (-0.16, -0.01) | 4.44E-02 | -0.11  (-0.22, 0.00) | 6.48E-02 |  | -0.05  (-0.20, 0.09) | 5.60E-01 | | -0.06  (-0.26, 0.14) | | 6.29E-01 | | |  |
| **Triglycerides concentration** |  |  |  |  |  |  |  | |  | |  | | |  |
| Total | -0.28  (-0.36, -0.20) | 2.65E-10 | -0.51  (-0.62, -0.39) | 5.60E-16 |  | -0.37  (-0.52, -0.21) | 5.80E-05 | | -0.46  (-0.66, -0.25) | | 3.25E-04 | | |  |
| VLDL | -0.27  (-0.35, -0.19) | 2.93E-10 | -0.50  (-0.61, -0.39) | 5.60E-16 |  | -0.35  (-0.50, -0.20) | 6.14E-05 | | -0.45  (-0.65, -0.25) | | 3.25E-04 | | |  |
| LDL | -0.20  (-0.28, -0.12) | 1.53E-06 | -0.31  (-0.42, -0.20) | 1.51E-07 |  | -0.26  (-0.41, -0.11) | 2.34E-03 | | -0.26  (-0.46, -0.06) | | 2.43E-02 | | |  |
| HDL | -0.20  (-0.28, -0.12) | 1.20E-06 | -0.35  (-0.46, -0.24) | 2.76E-09 |  | -0.27  (-0.42, -0.12) | 1.57E-03 | | -0.28  (-0.48, -0.08) | | 1.67E-02 | | |  |
| **Apolipoproteins** |  |  |  |  |  |  |  | |  | |  | | |  |
| Apolipoproteins A1 | 0.11  (0.03, 0.19) | 1.20E-02 | 0.12  (0.00, 0.23) | 6.10E-02 |  | 0.09  (-0.06, 0.24) | 3.03E-01 | | 0.10  (-0.10, 0.30) | | 4.13E-01 | | |  |
| Apolipoproteins B | -0.22  (-0.3, -0.15) | 4.73E-08 | -0.37  (-0.48, -0.25) | 3.36E-10 |  | -0.32  (-0.46, -0.17) | 1.34E-04 | | -0.32  (-0.52, -0.13) | | 3.61E-03 | | |  |
| Ratio: ApoB to ApoA1 | -0.27  (-0.35, -0.19) | 2.93E-10 | -0.42  (-0.53, -0.30) | 1.15E-12 |  | -0.35  (-0.50, -0.20) | 5.80E-05 | | -0.37  (-0.57, -0.18) | | 9.18E-04 | | |  |

CHD = coronary heart disease; CI = confidence interval; FDR = false discovery rate; VLDL = very low-density lipoprotein; IDL = intermediate-density lipoprotein; LDL = low-density lipoprotein; HDL = high-density lipoprotein; ApoA1 = apolipoprotein A1; ApoB = apolipoprotein B. Beta and 95% CI are for comparison of participants who adopted 2-3 or 4-5 healthy lifestyles with participants who adopted 0-1.

* Basic model was adjusted for: age, sex, fasting time, study sites, education level, and case/control status.

# Supplementary file 2C. Associations of the *HMGCR* score with changes in the lipid metabolites, and subgroup analysis of the association between healthy lifestyle and lipid metabolites according to *HMGCR* score

| **Metabolomics** | **GRS based on Chinese vs metabolites** | | **GRS based on European vs metabolites** | |  | **Healthy lifestyle vs metabolites** | | | |  | **FDR for interaction** |
| --- | --- | --- | --- | --- | --- | --- | --- | --- | --- | --- | --- |
|  |  |  |  |  |  | **High risk group*** | | **Low risk group*** | |  |  |
|  | **Beta** | **FDR** | **Beta** | **FDR** |  | **Beta** | **P value** | **Beta** | **FDR** |  |  |
| **Lipoprotein Particle Concentration** | | |  | |  | | |  | |  | |
| **VLDL** |  |  |  |  |  |  |  |  |  |  |  |
| Extremely large | -0.050 | 0.164 | -0.048 | 0.251 |  | -0.196 | 5.67E-16 | -0.168 | 6.06E-13 |  | 0.999 |
| Very large | -0.042 | 0.242 | -0.030 | 0.472 |  | -0.199 | 1.82E-16 | -0.176 | 7.28E-14 |  | 0.999 |
| Large | -0.042 | 0.244 | -0.028 | 0.507 |  | -0.208 | 5.17E-17 | -0.179 | 6.80E-14 |  | 0.999 |
| Medium | -0.048 | 0.169 | -0.032 | 0.444 |  | -0.209 | 3.30E-17 | -0.175 | 2.25E-13 |  | 0.999 |
| Small | -0.074 | 0.027 | -0.056 | 0.159 |  | -0.201 | 7.33E-17 | -0.158 | 3.08E-11 |  | 0.999 |
| Very small | -0.114 | 0.001 | -0.094 | 0.004 |  | -0.122 | 1.74E-07 | -0.068 | 4.38E-03 |  | 0.999 |
| **IDL** | -0.114 | 0.001 | -0.108 | 0.001 |  | -0.052 | 2.72E-02 | -0.016 | 5.60E-01 |  | 0.999 |
| **LDL** |  |  |  |  |  |  |  |  |  |  |  |
| Large | -0.112 | 0.001 | -0.112 | 0.001 |  | -0.051 | 3.01E-02 | -0.018 | 5.23E-01 |  | 0.999 |
| Medium | -0.109 | 0.001 | -0.110 | 0.001 |  | -0.053 | 2.48E-02 | -0.021 | 4.54E-01 |  | 0.999 |
| Small | -0.109 | 0.001 | -0.110 | 0.001 |  | -0.061 | 8.67E-03 | -0.027 | 2.90E-01 |  | 0.999 |
| **HDL** |  |  |  |  |  |  |  |  |  |  |  |
| Very large | -0.014 | 0.664 | -0.010 | 0.776 |  | 0.068 | 4.66E-03 | 0.082 | 6.73E-04 |  | 0.999 |
| Large | 0.034 | 0.293 | 0.030 | 0.472 |  | 0.147 | 1.16E-10 | 0.128 | 6.00E-08 |  | 0.999 |
| Medium | 0.022 | 0.514 | 0.012 | 0.776 |  | 0.055 | 2.51E-02 | 0.014 | 6.39E-01 |  | 0.999 |
| Small | -0.003 | 0.925 | -0.010 | 0.776 |  | -0.034 | 1.62E-01 | -0.072 | 2.94E-03 |  | 0.999 |
| **Mean particle diameter** | |  |  |  |  |  |  |  |  |  |  |
| VLDL | -0.008 | 0.806 | -0.002 | 0.968 |  | -0.174 | 2.44E-12 | -0.165 | 1.71E-12 |  | 0.999 |
| LDL | 0.031 | 0.293 | 0.032 | 0.393 |  | 0.031 | 1.62E-01 | 0.045 | 4.00E-02 |  | 0.999 |
| HDL | 0.019 | 0.564 | 0.016 | 0.663 |  | 0.118 | 2.45E-07 | 0.114 | 1.22E-06 |  | 0.999 |
| **Apolipoproteins** |  |  |  |  |  |  |  |  |  |  |  |
| Apolipoproteins A1 | -0.020 | 0.561 | -0.024 | 0.524 |  | 0.052 | 3.16E-02 | 0.036 | 1.68E-01 |  | 0.999 |
| Apolipoproteins B | -0.103 | 0.001 | -0.094 | 0.004 |  | -0.167 | 3.23E-13 | -0.117 | 7.54E-07 |  | 0.999 |
| Ratio: ApoB to ApoA1 | -0.093 | 0.004 | -0.080 | 0.017 |  | -0.187 | 3.26E-16 | -0.133 | 2.22E-08 |  | 0.999 |
| **Cholesterol Concentration** | |  |  |  |  |  |  |  |  |  |  |
| Total | -0.106 | 0.001 | -0.106 | 0.001 |  | -0.037 | 1.21E-01 | -0.011 | 7.04E-01 |  | 0.999 |
| VLDL | -0.089 | 0.007 | -0.070 | 0.050 |  | -0.192 | 4.97E-16 | -0.143 | 1.55E-09 |  | 0.999 |
| Remnant | -0.110 | 0.001 | -0.096 | 0.003 |  | -0.149 | 1.06E-10 | -0.101 | 1.81E-05 |  | 0.999 |
| LDL | -0.107 | 0.001 | -0.112 | 0.001 |  | -0.029 | 2.22E-01 | -0.003 | 9.28E-01 |  | 0.999 |
| HDL | 0.009 | 0.792 | 0.002 | 0.946 |  | 0.139 | 4.87E-09 | 0.111 | 4.33E-06 |  | 0.999 |
| HDL2 | 0.014 | 0.664 | 0.008 | 0.823 |  | 0.142 | 2.21E-09 | 0.112 | 3.57E-06 |  | 0.999 |
| HDL3 | -0.048 | 0.164 | -0.044 | 0.285 |  | 0.065 | 7.36E-03 | 0.072 | 3.01E-03 |  | 0.999 |
| Esterified | -0.104 | 0.001 | -0.110 | 0.001 |  | -0.024 | 2.96E-01 | -0.006 | 8.73E-01 |  | 0.999 |
| Free | -0.109 | 0.001 | -0.096 | 0.003 |  | -0.068 | 4.09E-03 | -0.019 | 5.03E-01 |  | 0.999 |
| **Within size-specific lipoprotein** | | |  |  |  |  |  |  |  |  |  |
| **VLDL** |  |  |  |  |  |  |  |  |  |  |  |
| Extremely large | -0.052 | 0.155 | -0.048 | 0.251 |  | -0.199 | 1.73E-16 | -0.164 | 1.53E-12 |  | 0.999 |
| Very large | -0.049 | 0.164 | -0.038 | 0.377 |  | -0.197 | 2.29E-16 | -0.169 | 3.15E-13 |  | 0.999 |
| Large | -0.049 | 0.164 | -0.034 | 0.430 |  | -0.206 | 6.03E-17 | -0.173 | 2.25E-13 |  | 0.999 |
| Medium | -0.066 | 0.054 | -0.048 | 0.251 |  | -0.205 | 5.23E-17 | -0.162 | 1.03E-11 |  | 0.999 |
| Small | -0.105 | 0.001 | -0.090 | 0.007 |  | -0.138 | 2.39E-09 | -0.090 | 1.62E-04 |  | 0.999 |
| Very small | -0.115 | 0.001 | -0.106 | 0.001 |  | -0.052 | 2.72E-02 | -0.012 | 6.64E-01 |  | 0.999 |
| **IDL** | -0.111 | 0.001 | -0.112 | 0.001 |  | -0.030 | 1.98E-01 | 0.002 | 9.33E-01 |  | 0.999 |
| **LDL** |  |  |  |  |  |  |  |  |  |  |  |
| Large | -0.108 | 0.001 | -0.114 | 0.001 |  | -0.032 | 1.71E-01 | -0.003 | 9.31E-01 |  | 0.999 |
| Medium | -0.103 | 0.001 | -0.110 | 0.001 |  | -0.026 | 2.58E-01 | -0.001 | 9.82E-01 |  | 0.999 |
| Small | -0.103 | 0.001 | -0.110 | 0.001 |  | -0.021 | 3.52E-01 | 0.005 | 8.73E-01 |  | 0.999 |
| **HDL** |  |  |  |  |  |  |  |  |  |  |  |
| Very large | -0.034 | 0.293 | -0.022 | 0.577 |  | 0.019 | 4.17E-01 | 0.045 | 7.32E-02 |  | 0.999 |
| Large | 0.039 | 0.248 | 0.036 | 0.377 |  | 0.148 | 9.51E-11 | 0.134 | 1.84E-08 |  | 0.999 |
| Medium | 0.033 | 0.299 | 0.018 | 0.663 |  | 0.096 | 7.02E-05 | 0.052 | 3.74E-02 |  | 0.999 |
| Small | -0.037 | 0.269 | -0.054 | 0.159 |  | 0.074 | 1.53E-03 | 0.056 | 1.70E-02 |  | 0.999 |
| **Triglycerides Concentration** | |  |  |  |  |  |  |  |  |  |  |
| Total | -0.050 | 0.164 | -0.026 | 0.507 |  | -0.217 | 1.22E-17 | -0.179 | 6.80E-14 |  | 0.999 |
| VLDL | -0.044 | 0.223 | -0.026 | 0.507 |  | -0.211 | 3.29E-17 | -0.181 | 6.80E-14 |  | 0.999 |
| LDL | -0.084 | 0.009 | -0.044 | 0.270 |  | -0.144 | 9.58E-10 | -0.097 | 3.98E-05 |  | 0.999 |
| HDL | -0.034 | 0.293 | -0.012 | 0.776 |  | -0.151 | 2.68E-10 | -0.122 | 1.63E-07 |  | 0.999 |
| **Within size-specific lipoprotein** | | |  |  |  |  |  |  |  |  |  |
| **VLDL** |  |  |  |  |  |  |  |  |  |  |  |
| Extremely large | -0.049 | 0.164 | -0.046 | 0.251 |  | -0.195 | 6.62E-16 | -0.167 | 6.06E-13 |  | 0.999 |
| Very large | -0.039 | 0.261 | -0.026 | 0.507 |  | -0.199 | 2.09E-16 | -0.178 | 6.80E-14 |  | 0.999 |
| Large | -0.039 | 0.262 | -0.026 | 0.513 |  | -0.208 | 5.23E-17 | -0.180 | 6.80E-14 |  | 0.999 |
| Medium | -0.041 | 0.248 | -0.026 | 0.507 |  | -0.208 | 5.13E-17 | -0.178 | 1.09E-13 |  | 0.999 |
| Small | -0.053 | 0.152 | -0.034 | 0.418 |  | -0.211 | 2.29E-17 | -0.175 | 2.25E-13 |  | 0.999 |
| Very small | -0.067 | 0.048 | -0.032 | 0.441 |  | -0.201 | 9.21E-17 | -0.146 | 8.12E-10 |  | 0.999 |
| **IDL** | -0.077 | 0.018 | -0.036 | 0.394 |  | -0.153 | 1.07E-10 | -0.104 | 9.15E-06 |  | 0.999 |
| **LDL** |  |  |  |  |  |  |  |  |  |  |  |
| Large | -0.081 | 0.012 | -0.042 | 0.285 |  | -0.126 | 7.46E-08 | -0.089 | 1.62E-04 |  | 0.999 |
| Medium | -0.082 | 0.012 | -0.058 | 0.139 |  | -0.125 | 1.17E-07 | -0.073 | 2.23E-03 |  | 0.999 |
| Small | -0.073 | 0.028 | -0.056 | 0.159 |  | -0.188 | 3.22E-15 | -0.128 | 7.08E-08 |  | 0.999 |
| **HDL** |  |  |  |  |  |  |  |  |  |  |  |
| Very large | -0.026 | 0.430 | -0.010 | 0.776 |  | -0.131 | 1.70E-08 | -0.091 | 1.01E-04 |  | 0.999 |
| Large | 0.001 | 0.970 | 0.010 | 0.776 |  | 0.024 | 2.74E-01 | 0.035 | 1.43E-01 |  | 0.999 |
| Medium | -0.038 | 0.262 | -0.028 | 0.488 |  | -0.158 | 1.03E-10 | -0.137 | 1.20E-08 |  | 0.999 |
| Small | -0.034 | 0.298 | -0.010 | 0.776 |  | -0.202 | 1.73E-16 | -0.174 | 3.12E-13 |  | 0.999 |

HMGCR = 3-hydroxy-3-methylglutaryl–coenzyme A reductase; GRS = genetic risk score; FDR = false discovery rate; VLDL =very low-density lipoprotein; IDL = intermediate-density lipoprotein; LDL = low-density lipoprotein; HDL = high-density lipoprotein; ApoA1 = apolipoprotein A1; ApoB = apolipoprotein B. Beta and 95% CI are for associations between *HMGCR* score and lipid markers. Models were adjusted for age, sex, survey sites, and the first 10 principle components.

*Participants were classified into two subgroups using median cutoffs of GRS based on Chinese population.

# Supplementary file 2D. Associations of the *ACLY* score with changes in the lipid metabolites, and subgroup analysis of the association between healthy lifestyle and lipid metabolites according to *ACLY* score

| **Metabolomics** | **GRS based on Chinese vs metabolites** | | **GRS based on European vs metabolites** | |  | **Healthy lifestyle vs metabolites** | | | |  | **FDR for interaction** |
| --- | --- | --- | --- | --- | --- | --- | --- | --- | --- | --- | --- |
|  |  |  |  |  |  | **High risk group*** | | **Low risk group*** | |  |  |
|  | **Beta** | **FDR** | **Beta** | **FDR** |  | **Beta** | **P value** | **Beta** | **FDR** |  |  |
| **Lipoprotein Particle Concentration** | | |  | |  | | |  | |  | |
| **VLDL** |  |  |  |  |  |  |  |  |  |  |  |
| Extremely large | -0.011 | 0.873 | -0.030 | 0.414 |  | -0.182 | 1.01E-14 | -0.182 | 5.67E-14 |  | 0.996 |
| Very large | -0.008 | 0.873 | -0.034 | 0.376 |  | -0.193 | 3.08E-16 | -0.183 | 3.41E-14 |  | 0.996 |
| Large | -0.012 | 0.873 | -0.034 | 0.378 |  | -0.198 | 2.01E-16 | -0.190 | 1.64E-14 |  | 0.996 |
| Medium | -0.017 | 0.865 | -0.042 | 0.348 |  | -0.200 | 2.01E-16 | -0.186 | 3.11E-14 |  | 0.996 |
| Small | -0.029 | 0.673 | -0.042 | 0.348 |  | -0.195 | 5.53E-16 | -0.166 | 3.88E-12 |  | 0.996 |
| Very small | -0.050 | 0.255 | -0.060 | 0.128 |  | -0.107 | 6.06E-06 | -0.082 | 4.04E-04 |  | 0.996 |
| **IDL** | -0.067 | 0.093 | -0.074 | 0.061 |  | -0.052 | 2.98E-02 | -0.014 | 6.13E-01 |  | 0.996 |
| **LDL** |  |  |  |  |  |  |  |  |  |  |  |
| Large | -0.073 | 0.063 | -0.074 | 0.061 |  | -0.056 | 1.92E-02 | -0.010 | 6.86E-01 |  | 0.996 |
| Medium | -0.075 | 0.062 | -0.072 | 0.061 |  | -0.061 | 1.02E-02 | -0.010 | 6.86E-01 |  | 0.996 |
| Small | -0.080 | 0.062 | -0.074 | 0.061 |  | -0.069 | 3.60E-03 | -0.017 | 5.42E-01 |  | 0.996 |
| **HDL** |  |  |  |  |  |  |  |  |  |  |  |
| Very large | -0.025 | 0.767 | -0.026 | 0.468 |  | 0.092 | 1.02E-04 | 0.060 | 1.47E-02 |  | 0.996 |
| Large | 0.011 | 0.873 | 0.004 | 0.906 |  | 0.154 | 2.43E-11 | 0.122 | 2.16E-07 |  | 0.996 |
| Medium | -0.002 | 0.970 | 0.000 | 0.990 |  | 0.035 | 1.48E-01 | 0.035 | 1.87E-01 |  | 0.996 |
| Small | -0.023 | 0.820 | -0.008 | 0.859 |  | -0.065 | 7.25E-03 | -0.041 | 1.05E-01 |  | 0.996 |
| **Mean particle diameter** | |  |  |  |  |  |  |  |  |  |  |
| VLDL | 0.003 | 0.970 | -0.024 | 0.540 |  | -0.171 | 8.05E-13 | -0.169 | 3.93E-12 |  | 0.996 |
| LDL | 0.045 | 0.255 | 0.016 | 0.656 |  | 0.062 | 4.12E-03 | 0.012 | 6.37E-01 |  | 0.996 |
| HDL | 0.001 | 0.972 | -0.008 | 0.859 |  | 0.136 | 3.68E-09 | 0.097 | 3.22E-05 |  | 0.996 |
| **Apolipoproteins** |  |  |  |  |  |  |  |  |  |  |  |
| Apolipoproteins A1 | -0.037 | 0.427 | -0.034 | 0.378 |  | 0.038 | 1.15E-01 | 0.054 | 3.33E-02 |  | 0.996 |
| Apolipoproteins B | -0.065 | 0.101 | -0.078 | 0.061 |  | -0.163 | 4.80E-12 | -0.121 | 1.65E-07 |  | 0.996 |
| Ratio: ApoB to ApoA1 | -0.043 | 0.356 | -0.056 | 0.186 |  | -0.178 | 2.14E-14 | -0.143 | 1.10E-09 |  | 0.996 |
| **Cholesterol Concentration** | |  |  |  |  |  |  |  |  |  |  |
| Total | -0.076 | 0.062 | -0.072 | 0.061 |  | -0.044 | 6.59E-02 | -0.001 | 9.67E-01 |  | 0.996 |
| VLDL | -0.039 | 0.400 | -0.060 | 0.138 |  | -0.179 | 4.37E-14 | -0.158 | 3.33E-11 |  | 0.996 |
| Remnant | -0.062 | 0.124 | -0.074 | 0.061 |  | -0.141 | 1.88E-09 | -0.108 | 3.12E-06 |  | 0.996 |
| LDL | -0.075 | 0.062 | -0.070 | 0.076 |  | -0.041 | 8.57E-02 | 0.011 | 6.82E-01 |  | 0.996 |
| HDL | -0.020 | 0.865 | -0.008 | 0.859 |  | 0.132 | 2.54E-08 | 0.120 | 7.04E-07 |  | 0.996 |
| HDL2 | -0.015 | 0.865 | -0.004 | 0.906 |  | 0.135 | 1.27E-08 | 0.121 | 5.79E-07 |  | 0.996 |
| HDL3 | -0.058 | 0.170 | -0.050 | 0.234 |  | 0.062 | 1.05E-02 | 0.079 | 1.11E-03 |  | 0.996 |
| Esterified | -0.077 | 0.062 | -0.080 | 0.061 |  | -0.033 | 1.48E-01 | 0.006 | 8.07E-01 |  | 0.996 |
| Free | -0.065 | 0.101 | -0.046 | 0.275 |  | -0.066 | 5.23E-03 | -0.017 | 5.42E-01 |  | 0.996 |
| **Within size-specific lipoprotein** | | |  |  |  |  |  |  |  |  |  |
| **VLDL** |  |  |  |  |  |  |  |  |  |  |  |
| Extremely large | -0.007 | 0.873 | -0.040 | 0.349 |  | -0.183 | 4.68E-15 | -0.180 | 6.97E-14 |  | 0.996 |
| Very large | -0.007 | 0.873 | -0.038 | 0.349 |  | -0.188 | 8.27E-16 | -0.179 | 6.97E-14 |  | 0.996 |
| Large | -0.010 | 0.873 | -0.034 | 0.378 |  | -0.194 | 4.94E-16 | -0.187 | 3.11E-14 |  | 0.996 |
| Medium | -0.019 | 0.865 | -0.046 | 0.288 |  | -0.191 | 1.18E-15 | -0.177 | 3.10E-13 |  | 0.996 |
| Small | -0.048 | 0.269 | -0.050 | 0.238 |  | -0.132 | 1.92E-08 | -0.097 | 3.61E-05 |  | 0.996 |
| Very small | -0.054 | 0.214 | -0.066 | 0.089 |  | -0.043 | 7.05E-02 | -0.020 | 4.71E-01 |  | 0.996 |
| **IDL** | -0.072 | 0.063 | -0.074 | 0.061 |  | -0.032 | 1.63E-01 | 0.006 | 7.99E-01 |  | 0.996 |
| **LDL** |  |  |  |  |  |  |  |  |  |  |  |
| Large | -0.075 | 0.062 | -0.072 | 0.061 |  | -0.040 | 9.17E-02 | 0.007 | 7.91E-01 |  | 0.996 |
| Medium | -0.074 | 0.062 | -0.068 | 0.080 |  | -0.037 | 1.08E-01 | 0.013 | 6.37E-01 |  | 0.996 |
| Small | -0.076 | 0.062 | -0.066 | 0.089 |  | -0.031 | 1.70E-01 | 0.018 | 5.40E-01 |  | 0.996 |
| **HDL** |  |  |  |  |  |  |  |  |  |  |  |
| Very large | -0.039 | 0.400 | -0.040 | 0.349 |  | 0.044 | 6.91E-02 | 0.024 | 4.12E-01 |  | 0.996 |
| Large | 0.013 | 0.873 | 0.006 | 0.884 |  | 0.160 | 5.20E-12 | 0.123 | 1.88E-07 |  | 0.996 |
| Medium | -0.006 | 0.898 | 0.006 | 0.887 |  | 0.074 | 2.23E-03 | 0.074 | 2.79E-03 |  | 0.996 |
| Small | -0.050 | 0.255 | -0.016 | 0.664 |  | 0.048 | 3.74E-02 | 0.083 | 4.28E-04 |  | 0.996 |
| **Triglycerides Concentration** | |  |  |  |  |  |  |  |  |  |  |
| Total | -0.017 | 0.865 | -0.042 | 0.348 |  | -0.204 | 1.16E-16 | -0.194 | 1.21E-14 |  | 0.996 |
| VLDL | -0.016 | 0.865 | -0.038 | 0.349 |  | -0.203 | 1.16E-16 | -0.190 | 1.64E-14 |  | 0.996 |
| LDL | -0.041 | 0.387 | -0.052 | 0.229 |  | -0.124 | 2.24E-07 | -0.118 | 3.70E-07 |  | 0.996 |
| HDL | 0.007 | 0.873 | -0.024 | 0.513 |  | -0.135 | 8.35E-09 | -0.138 | 6.62E-09 |  | 0.996 |
| **Within size-specific lipoprotein** | | |  |  |  |  |  |  |  |  |  |
| **VLDL** |  |  |  |  |  |  |  |  |  |  |  |
| Extremely large | -0.009 | 0.873 | -0.028 | 0.468 |  | -0.180 | 1.36E-14 | -0.182 | 5.50E-14 |  | 0.996 |
| Very large | -0.008 | 0.873 | -0.034 | 0.378 |  | -0.194 | 2.54E-16 | -0.184 | 3.24E-14 |  | 0.996 |
| Large | -0.013 | 0.873 | -0.032 | 0.382 |  | -0.199 | 2.01E-16 | -0.190 | 1.64E-14 |  | 0.996 |
| Medium | -0.016 | 0.865 | -0.038 | 0.350 |  | -0.200 | 2.01E-16 | -0.187 | 3.11E-14 |  | 0.996 |
| Small | -0.019 | 0.865 | -0.032 | 0.382 |  | -0.208 | 6.73E-17 | -0.180 | 6.97E-14 |  | 0.996 |
| Very small | -0.017 | 0.865 | -0.036 | 0.373 |  | -0.185 | 1.64E-14 | -0.165 | 4.03E-12 |  | 0.996 |
| **IDL** | -0.026 | 0.724 | -0.036 | 0.350 |  | -0.135 | 1.51E-08 | -0.124 | 1.12E-07 |  | 0.996 |
| **LDL** |  |  |  |  |  |  |  |  |  |  |  |
| Large | -0.040 | 0.387 | -0.044 | 0.288 |  | -0.114 | 1.98E-06 | -0.101 | 1.06E-05 |  | 0.996 |
| Medium | -0.048 | 0.269 | -0.042 | 0.328 |  | -0.105 | 1.26E-05 | -0.094 | 5.28E-05 |  | 0.996 |
| Small | -0.043 | 0.359 | -0.056 | 0.188 |  | -0.171 | 1.66E-12 | -0.146 | 5.00E-10 |  | 0.996 |
| **HDL** |  |  |  |  |  |  |  |  |  |  |  |
| Very large | -0.009 | 0.873 | -0.040 | 0.349 |  | -0.111 | 1.38E-06 | -0.108 | 4.65E-06 |  | 0.996 |
| Large | 0.015 | 0.865 | -0.010 | 0.842 |  | 0.037 | 9.90E-02 | 0.024 | 3.50E-01 |  | 0.996 |
| Medium | 0.008 | 0.873 | -0.018 | 0.664 |  | -0.148 | 6.73E-10 | -0.148 | 1.24E-09 |  | 0.996 |
| Small | -0.002 | 0.970 | -0.022 | 0.540 |  | -0.195 | 7.70E-16 | -0.183 | 5.50E-14 |  | 0.996 |

ACLY = ATP citrate lyase; GRS = genetic risk score; FDR = false discovery rate; VLDL =very low-density lipoprotein; IDL = intermediate-density lipoprotein; LDL = low-density lipoprotein; HDL = high-density lipoprotein; ApoA1 = apolipoprotein A1; ApoB = apolipoprotein B. Beta and 95% CI are for associations between *ACLY* score and lipid markers. Models were adjusted for age, sex, survey sites, and the first 10 principle components.

*Participants were classified into two subgroups using median cutoffs of GRS based on Chinese population.

# Supplementary file 2E. Associations of the sum of *HMGCR* and *ACLY* score with changes in the lipid metabolites, and subgroup analysis of the association between healthy lifestyle and lipid metabolites according to the sum score

| **Metabolomics** | **GRS based on Chinese vs metabolites** | | **GRS based on European vs metabolites** | |  | **Healthy lifestyle vs metabolites** | | | |  | **FDR for interaction** |
| --- | --- | --- | --- | --- | --- | --- | --- | --- | --- | --- | --- |
|  |  |  |  |  |  | **High risk group*** | | **Low risk group*** | |  |  |
|  | **Beta** | **FDR** | **Beta** | **FDR** |  | **Beta** | **P value** | **Beta** | **FDR** |  |  |
| **Lipoprotein Particle Concentration** | | |  | |  | | |  | |  | |
| **VLDL** |  |  |  |  |  |  |  |  |  |  |  |
| Extremely large | -0.046 | 0.189 | -0.060 | 0.083 |  | -0.181 | 8.78E-14 | -0.181 | 4.72E-15 |  | 0.973 |
| Very large | -0.038 | 0.253 | -0.046 | 0.174 |  | -0.187 | 1.61E-14 | -0.188 | 8.69E-16 |  | 0.973 |
| Large | -0.041 | 0.228 | -0.044 | 0.201 |  | -0.196 | 2.85E-15 | -0.191 | 8.69E-16 |  | 0.973 |
| Medium | -0.050 | 0.163 | -0.052 | 0.129 |  | -0.196 | 2.70E-15 | -0.189 | 1.75E-15 |  | 0.973 |
| Small | -0.079 | 0.016 | -0.076 | 0.024 |  | -0.182 | 4.24E-14 | -0.177 | 6.72E-14 |  | 0.973 |
| Very small | -0.126 | <0.001 | -0.122 | <0.001 |  | -0.090 | 1.33E-04 | -0.097 | 2.97E-05 |  | 0.973 |
| **IDL** | -0.136 | <0.001 | -0.142 | <0.001 |  | -0.029 | 2.29E-01 | -0.036 | 1.45E-01 |  | 0.973 |
| **LDL** |  |  |  |  |  |  |  |  |  |  |  |
| Large | -0.137 | <0.001 | -0.146 | <0.001 |  | -0.032 | 1.79E-01 | -0.033 | 1.82E-01 |  | 0.973 |
| Medium | -0.135 | <0.001 | -0.146 | <0.001 |  | -0.038 | 1.13E-01 | -0.033 | 1.86E-01 |  | 0.973 |
| Small | -0.139 | <0.001 | -0.146 | <0.001 |  | -0.047 | 4.56E-02 | -0.038 | 1.25E-01 |  | 0.973 |
| **HDL** |  |  |  |  |  |  |  |  |  |  |  |
| Very large | -0.027 | 0.404 | -0.012 | 0.712 |  | 0.081 | 6.93E-04 | 0.072 | 2.91E-03 |  | 0.973 |
| Large | 0.034 | 0.280 | 0.036 | 0.265 |  | 0.141 | 7.38E-10 | 0.136 | 7.99E-09 |  | 0.973 |
| Medium | 0.021 | 0.530 | 0.018 | 0.604 |  | 0.040 | 1.13E-01 | 0.030 | 2.34E-01 |  | 0.973 |
| Small | -0.013 | 0.692 | -0.012 | 0.713 |  | -0.053 | 2.97E-02 | -0.052 | 3.39E-02 |  | 0.978 |
| **Mean particle diameter** | |  |  |  |  |  |  |  |  |  |  |
| VLDL | -0.004 | 0.914 | -0.012 | 0.729 |  | -0.171 | 3.95E-12 | -0.169 | 6.51E-13 |  | 0.973 |
| LDL | 0.048 | 0.117 | 0.044 | 0.138 |  | 0.049 | 2.45E-02 | 0.026 | 2.48E-01 |  | 0.973 |
| HDL | 0.016 | 0.629 | 0.020 | 0.551 |  | 0.123 | 9.35E-08 | 0.111 | 1.76E-06 |  | 0.973 |
| **Apolipoproteins** |  |  |  |  |  |  |  |  |  |  |  |
| Apolipoproteins A1 | -0.038 | 0.254 | -0.036 | 0.269 |  | 0.049 | 4.74E-02 | 0.042 | 9.89E-02 |  | 0.973 |
| Apolipoproteins B | -0.125 | <0.001 | -0.132 | <0.001 |  | -0.143 | 7.91E-10 | -0.140 | 1.83E-09 |  | 0.973 |
| Ratio: ApoB to ApoA1 | -0.103 | 0.001 | -0.110 | <0.001 |  | -0.163 | 1.95E-12 | -0.158 | 2.28E-11 |  | 0.973 |
| **Cholesterol Concentration** | |  |  |  |  |  |  |  |  |  |  |
| Total | -0.134 | <0.001 | -0.140 | <0.001 |  | -0.020 | 4.25E-01 | -0.024 | 3.19E-01 |  | 0.973 |
| VLDL | -0.098 | 0.002 | -0.100 | 0.002 |  | -0.164 | 5.66E-12 | -0.171 | 3.05E-13 |  | 0.973 |
| Remnant | -0.130 | <0.001 | -0.132 | <0.001 |  | -0.120 | 2.73E-07 | -0.128 | 3.05E-08 |  | 0.973 |
| LDL | -0.134 | <0.001 | -0.146 | <0.001 |  | -0.014 | 5.61E-01 | -0.015 | 5.31E-01 |  | 0.973 |
| HDL | -0.003 | 0.920 | 0.004 | 0.890 |  | 0.129 | 5.55E-08 | 0.124 | 2.66E-07 |  | 0.973 |
| HDL2 | 0.004 | 0.908 | 0.010 | 0.729 |  | 0.131 | 3.73E-08 | 0.126 | 1.65E-07 |  | 0.973 |
| HDL3 | -0.077 | 0.017 | -0.062 | 0.070 |  | 0.070 | 3.96E-03 | 0.071 | 3.44E-03 |  | 0.978 |
| Esterified | -0.133 | <0.001 | -0.148 | <0.001 |  | -0.006 | 8.02E-01 | -0.021 | 3.92E-01 |  | 0.999 |
| Free | -0.128 | <0.001 | -0.114 | <0.001 |  | -0.054 | 2.52E-02 | -0.029 | 2.37E-01 |  | 0.973 |
| **Within size-specific lipoprotein** | | |  |  |  |  |  |  |  |  |  |
| **VLDL** |  |  |  |  |  |  |  |  |  |  |  |
| Extremely large | -0.047 | 0.178 | -0.066 | 0.058 |  | -0.180 | 8.75E-14 | -0.183 | 2.89E-15 |  | 0.999 |
| Very large | -0.044 | 0.193 | -0.056 | 0.106 |  | -0.180 | 7.65E-14 | -0.186 | 1.26E-15 |  | 0.973 |
| Large | -0.046 | 0.189 | -0.050 | 0.141 |  | -0.191 | 9.47E-15 | -0.188 | 1.47E-15 |  | 0.973 |
| Medium | -0.066 | 0.050 | -0.070 | 0.040 |  | -0.185 | 2.93E-14 | -0.181 | 1.78E-14 |  | 0.973 |
| Small | -0.117 | <0.001 | -0.116 | <0.001 |  | -0.112 | 1.42E-06 | -0.115 | 9.75E-07 |  | 0.973 |
| Very small | -0.130 | <0.001 | -0.134 | <0.001 |  | -0.021 | 4.03E-01 | -0.041 | 9.35E-02 |  | 0.999 |
| **IDL** | -0.136 | <0.001 | -0.146 | <0.001 |  | -0.010 | 6.81E-01 | -0.015 | 5.31E-01 |  | 0.973 |
| **LDL** |  |  |  |  |  |  |  |  |  |  |  |
| Large | -0.135 | <0.001 | -0.148 | <0.001 |  | -0.016 | 5.24E-01 | -0.016 | 5.02E-01 |  | 0.973 |
| Medium | -0.130 | <0.001 | -0.144 | <0.001 |  | -0.013 | 5.83E-01 | -0.011 | 6.40E-01 |  | 0.973 |
| Small | -0.131 | <0.001 | -0.142 | <0.001 |  | -0.009 | 7.10E-01 | -0.004 | 8.44E-01 |  | 0.973 |
| **HDL** |  |  |  |  |  |  |  |  |  |  |  |
| Very large | -0.052 | 0.134 | -0.032 | 0.347 |  | 0.034 | 1.67E-01 | 0.034 | 1.87E-01 |  | 0.973 |
| Large | 0.040 | 0.224 | 0.044 | 0.180 |  | 0.142 | 5.63E-10 | 0.141 | 2.20E-09 |  | 0.973 |
| Medium | 0.028 | 0.399 | 0.026 | 0.455 |  | 0.076 | 1.63E-03 | 0.072 | 3.44E-03 |  | 0.973 |
| Small | -0.058 | 0.085 | -0.064 | 0.058 |  | 0.063 | 7.04E-03 | 0.068 | 3.44E-03 |  | 0.973 |
| **Triglycerides Concentration** | |  |  |  |  |  |  |  |  |  |  |
| Total | -0.051 | 0.148 | -0.048 | 0.169 |  | -0.201 | 2.17E-15 | -0.195 | 8.69E-16 |  | 0.973 |
| VLDL | -0.045 | 0.193 | -0.044 | 0.188 |  | -0.198 | 2.70E-15 | -0.194 | 8.69E-16 |  | 0.973 |
| LDL | -0.096 | 0.002 | -0.066 | 0.047 |  | -0.116 | 1.30E-06 | -0.122 | 1.22E-07 |  | 0.973 |
| HDL | -0.026 | 0.408 | -0.024 | 0.485 |  | -0.131 | 5.07E-08 | -0.142 | 8.77E-10 |  | 0.973 |
| **Within size-specific lipoprotein** | | |  |  |  |  |  |  |  |  |  |
| **VLDL** |  |  |  |  |  |  |  |  |  |  |  |
| Extremely large | -0.044 | 0.193 | -0.058 | 0.095 |  | -0.180 | 9.51E-14 | -0.181 | 4.91E-15 |  | 0.973 |
| Very large | -0.036 | 0.280 | -0.042 | 0.201 |  | -0.188 | 1.21E-14 | -0.188 | 8.69E-16 |  | 0.973 |
| Large | -0.039 | 0.253 | -0.040 | 0.230 |  | -0.197 | 2.70E-15 | -0.190 | 8.75E-16 |  | 0.973 |
| Medium | -0.043 | 0.216 | -0.044 | 0.188 |  | -0.197 | 2.70E-15 | -0.189 | 1.51E-15 |  | 0.973 |
| Small | -0.055 | 0.117 | -0.050 | 0.140 |  | -0.198 | 2.17E-15 | -0.188 | 2.12E-15 |  | 0.973 |
| Very small | -0.067 | 0.048 | -0.052 | 0.128 |  | -0.177 | 2.02E-13 | -0.169 | 6.51E-13 |  | 0.973 |
| **IDL** | -0.082 | 0.010 | -0.054 | 0.116 |  | -0.128 | 7.63E-08 | -0.127 | 4.21E-08 |  | 0.999 |
| **LDL** |  |  |  |  |  |  |  |  |  |  |  |
| Large | -0.093 | 0.003 | -0.062 | 0.065 |  | -0.103 | 1.57E-05 | -0.109 | 1.96E-06 |  | 0.973 |
| Medium | -0.100 | 0.002 | -0.076 | 0.024 |  | -0.101 | 2.44E-05 | -0.093 | 5.51E-05 |  | 0.999 |
| Small | -0.088 | 0.006 | -0.082 | 0.015 |  | -0.171 | 2.20E-12 | -0.143 | 8.32E-10 |  | 0.973 |
| **HDL** |  |  |  |  |  |  |  |  |  |  |  |
| Very large | -0.031 | 0.352 | -0.028 | 0.387 |  | -0.112 | 1.72E-06 | -0.109 | 2.40E-06 |  | 0.973 |
| Large | 0.005 | 0.889 | 0.006 | 0.827 |  | 0.037 | 1.09E-01 | 0.023 | 3.26E-01 |  | 0.973 |
| Medium | -0.027 | 0.404 | -0.036 | 0.288 |  | -0.142 | 6.07E-09 | -0.153 | 1.41E-10 |  | 0.999 |
| Small | -0.030 | 0.370 | -0.020 | 0.551 |  | -0.192 | 8.81E-15 | -0.184 | 6.02E-15 |  | 0.973 |

HMGCR = 3-hydroxy-3-methylglutaryl–coenzyme A reductase; ACLY = ATP citrate lyase; GRS = genetic risk score; FDR = false discovery rate; VLDL =very low-density lipoprotein; IDL = intermediate-density lipoprotein; LDL = low-density lipoprotein; HDL = high-density lipoprotein; ApoA1 = apolipoprotein A1; ApoB = apolipoprotein B. Beta and 95% CI are for associations between the sum score and lipid markers. Models were adjusted for age, sex, survey sites, and the first 10 principle components.

*Participants were classified into two subgroups using median cutoffs of GRS based on Chinese population.

# Supplementary file 2F. Associations of the genetic scores* with changes in the lipid metabolites

| **Metabolomics** | ***HMGCR* score vs metabolites** | |  | ***ACLY* score vs metabolites** | |
| --- | --- | --- | --- | --- | --- |
|  | **Beta** | **FDR** |  | **Beta** | **FDR** |
| **Lipoprotein Particle Concentration** | | |  |  |  |
| **VLDL** |  |  |  |  |  |
| Extremely large | -0.046 | 0.279 |  | -0.040 | 0.425 |
| Very large | -0.030 | 0.476 |  | -0.044 | 0.425 |
| Large | -0.026 | 0.529 |  | -0.042 | 0.425 |
| Medium | -0.030 | 0.476 |  | -0.042 | 0.425 |
| Small | -0.054 | 0.184 |  | -0.030 | 0.453 |
| Very small | -0.088 | 0.008 |  | -0.034 | 0.450 |
| **IDL** | -0.102 | 0.002 |  | -0.040 | 0.425 |
| **LDL** |  |  |  |  |  |
| Large | -0.104 | 0.002 |  | -0.038 | 0.425 |
| Medium | -0.104 | 0.002 |  | -0.034 | 0.426 |
| Small | -0.104 | 0.002 |  | -0.036 | 0.426 |
| **HDL** |  |  |  |  |  |
| Very large | -0.008 | 0.836 |  | -0.030 | 0.453 |
| Large | 0.028 | 0.476 |  | -0.004 | 0.948 |
| Medium | 0.008 | 0.836 |  | -0.008 | 0.896 |
| Small | -0.014 | 0.772 |  | -0.002 | 0.948 |
| **Mean particle diameter** | |  |  |  |  |
| VLDL | -0.002 | 0.955 |  | -0.040 | 0.425 |
| LDL | 0.032 | 0.418 |  | 0.006 | 0.900 |
| HDL | 0.018 | 0.655 |  | -0.014 | 0.736 |
| **Apolipoproteins** |  |  |  |  |  |
| Apolipoproteins A1 | -0.024 | 0.541 |  | -0.030 | 0.453 |
| Apolipoproteins B | -0.088 | 0.008 |  | -0.052 | 0.425 |
| Ratio: ApoB to ApoA1 | -0.076 | 0.029 |  | -0.034 | 0.450 |
| **Cholesterol Concentration** | |  |  |  |  |
| Total | -0.102 | 0.002 |  | -0.042 | 0.425 |
| VLDL | -0.066 | 0.069 |  | -0.046 | 0.425 |
| Remnant | -0.092 | 0.006 |  | -0.048 | 0.425 |
| LDL | -0.106 | 0.002 |  | -0.032 | 0.453 |
| HDL | 0.002 | 0.952 |  | -0.010 | 0.820 |
| HDL2 | 0.006 | 0.842 |  | -0.006 | 0.900 |
| HDL3 | -0.040 | 0.364 |  | -0.038 | 0.425 |
| Esterified | -0.104 | 0.002 |  | -0.052 | 0.425 |
| Free | -0.090 | 0.006 |  | -0.016 | 0.652 |
| **Within size-specific lipoprotein** | | |  |  |  |
| **VLDL** |  |  |  |  |  |
| Extremely large | -0.046 | 0.279 |  | -0.048 | 0.425 |
| Very large | -0.036 | 0.400 |  | -0.048 | 0.425 |
| Large | -0.032 | 0.466 |  | -0.042 | 0.425 |
| Medium | -0.046 | 0.281 |  | -0.042 | 0.425 |
| Small | -0.086 | 0.010 |  | -0.022 | 0.565 |
| Very small | -0.100 | 0.002 |  | -0.034 | 0.426 |
| **IDL** | -0.106 | 0.002 |  | -0.040 | 0.425 |
| **LDL** |  |  |  |  |  |
| Large | -0.106 | 0.002 |  | -0.036 | 0.426 |
| Medium | -0.104 | 0.002 |  | -0.030 | 0.453 |
| Small | -0.102 | 0.002 |  | -0.028 | 0.473 |
| **HDL** |  |  |  |  |  |
| Very large | -0.020 | 0.632 |  | -0.042 | 0.425 |
| Large | 0.036 | 0.400 |  | -0.002 | 0.948 |
| Medium | 0.014 | 0.767 |  | -0.002 | 0.962 |
| Small | -0.054 | 0.183 |  | 0.018 | 0.652 |
| **Triglycerides Concentration** | |  |  |  |  |
| Total | -0.026 | 0.529 |  | -0.042 | 0.425 |
| VLDL | -0.024 | 0.529 |  | -0.042 | 0.425 |
| LDL | -0.040 | 0.362 |  | -0.032 | 0.453 |
| HDL | -0.010 | 0.802 |  | -0.030 | 0.453 |
| **Within size-specific lipoprotein** | | |  |  |  |
| **VLDL** |  |  |  |  |  |
| Extremely large | -0.046 | 0.279 |  | -0.040 | 0.425 |
| Very large | -0.026 | 0.529 |  | -0.042 | 0.425 |
| Large | -0.024 | 0.529 |  | -0.042 | 0.425 |
| Medium | -0.024 | 0.529 |  | -0.042 | 0.425 |
| Small | -0.034 | 0.459 |  | -0.032 | 0.453 |
| Very small | -0.030 | 0.476 |  | -0.024 | 0.552 |
| **IDL** | -0.032 | 0.476 |  | -0.020 | 0.620 |
| **LDL** |  |  |  |  |  |
| Large | -0.038 | 0.391 |  | -0.028 | 0.477 |
| Medium | -0.054 | 0.184 |  | -0.018 | 0.650 |
| Small | -0.052 | 0.184 |  | -0.036 | 0.426 |
| **HDL** |  |  |  |  |  |
| Very large | -0.008 | 0.838 |  | -0.050 | 0.425 |
| Large | 0.010 | 0.802 |  | -0.016 | 0.652 |
| Medium | -0.030 | 0.476 |  | -0.020 | 0.620 |
| Small | -0.010 | 0.823 |  | -0.026 | 0.507 |

HMGCR = 3-hydroxy-3-methylglutaryl–coenzyme A reductase; ACLY = ATP citrate lyase; FDR = false discovery rate; VLDL =very low-density lipoprotein; IDL = intermediate-density lipoprotein; LDL = low-density lipoprotein; HDL = high-density lipoprotein; ApoA1 = apolipoprotein A1; ApoB = apolipoprotein B. Beta and 95% CI are for associations between genetic scores and lipid markers. Models were adjusted for age, sex, survey sites, and the first 10 principle components.

*Genetic scores calculated using SNPs and coefficients from the previous study based on the European population.

# Supplementary file 2G Food frequency questionnaire used in the CKB study at baseline

**During the past 12 months, about how often did you eat the following foods?**

|  | **Daily** | **4-6 days/week** | **1-3 days/week** | **Monthly** | **Never/rarely** |
| --- | --- | --- | --- | --- | --- |
| Rice | □ | □ | □ | □ | □ |
| Wheat | □ | □ | □ | □ | □ |
| Other staple food (corn, millet, etc.) | □ | □ | □ | □ | □ |
| Meat | □ | □ | □ | □ | □ |
| Poultry | □ | □ | □ | □ | □ |
| Fish/sea food | □ | □ | □ | □ | □ |
| Fresh eggs | □ | □ | □ | □ | □ |
| Fresh vegetables | □ | □ | □ | □ | □ |
| Soybean products | □ | □ | □ | □ | □ |
| Preserved vegetables | □ | □ | □ | □ | □ |
| Fresh fruit | □ | □ | □ | □ | □ |
| Dairy products (milk, yogurt) | □ | □ | □ | □ | □ |

**Supplementary file 2H. Variants included in the genetic scores and their associations with plasma low-density lipoprotein cholesterol.**

|  | **Exposure allele** | **Beta** | **P value** | **Exposure allele frequency** | **Imputation R^2^** |
| --- | --- | --- | --- | --- | --- |
| ***HMGCR* score with variants selected in other 13,060 CKB participants*** | | | | | |
| rs10043960 | A | -0.06 | 0.0002 | 0.63 | 0.96 |
| rs150712818 | T | -0.08 | 0.0085 | 0.05 | 0.94 |
| rs4704204 | A | -0.06 | 0.0009 | 0.33 | 0.97 |
| rs4704227 | G | -0.10 | 3.5E-09 | 0.48 | 0.98 |
| rs717587 | C | -0.05 | 0.0301 | 0.15 | 0.99 |
| rs536899312 | C | -1.06 | 0.0318 | 0.99 | 0.90 |
| rs200915188 | A | -0.24 | 0.0136 | 0.01 | 0.90 |
| ***ACLY* score with variants selected in other 13,060 CKB participants*** | | | | | |
| rs12938332 | G | -0.04 | 0.0063 | 0.41 | 0.97 |
| rs2070671 | G | -0.06 | 0.0062 | 0.84 | 1.00 |
| rs4796739 | C | -0.03 | 0.0421 | 0.66 | 0.99 |
| rs58523053 | A | -0.07 | 0.0027 | 0.15 | 0.87 |
| rs760282 | A | -0.05 | 0.0028 | 0.47 | 0.91 |
| ***HMGCR* score with variants previously reported**† | | | | | |
| rs12916 | T | -0.08 | 3.5E-14 | 0.47 | 1.00 |
| rs17238484 | G | -0.08 | 1.1E-11 | 0.71 | 0.99 |
| rs5909 | G | -0.06 | 0.082 | 0.98 | 1.00 |
| rs2303152 | G | -0.05 | 0.011 | 0.09 | 0.99 |
| rs10066707 | G | -0.05 | 5.3E-05 | 0.52 | 0.94 |
| rs2006760 | C | -0.04 | 6.9E-04 | 0.73 | 0.90 |
| ***ACLY* score with variants previously reported**† | | | | | |
| rs34349578 | G | -0.02 | 0.110 | 0.74 | 0.93 |
| rs55674565 | T | -0.02 | 0.047 | 0.58 | 0.99 |
| rs2883233 | C | -0.10 | 0.130 | 0.01 | 0.36 |
| rs117335915 | T | -0.13 | 0.083 | 0.99 | 0.52 |
| rs62075782 | C | -0.05 | 0.030 | 0.02 | 0.40 |
| rs143382920 | A | -0.06 | 0.060 | 0.98 | 0.73 |

HMGCR = 3-hydroxy-3-methylglutaryl–coenzyme A reductase; ACLY = ATP citrate lyase; CKB = China Kadoorie Biobank.

* For variants selected in 13,060 CKB participants, we reported the conditional effect of that variant on low-density lipoprotein cholesterol level in mmol/L adjusted for age, sex, the top 10 ancestry-informative principal components, and all other variants included in the score among the 13,060 participants. The 13,060 CKB participants were those who did not use lipid lowering medications at baseline and were not in the 4,681 lipidomic study subjects.

† For previously reported variants, we reported the effect of each variant on low-density lipoprotein cholesterol level of the current population, with adjustment for age, sex, and the top 10 principal components.
